# Ozone and Other Air Pollutants and the Risk of Congenital Heart Defects

**DOI:** 10.1038/srep34852

**Published:** 2016-10-18

**Authors:** Bin Zhang, Jinzhu Zhao, Rong Yang, Zhengmin Qian, Shengwen Liang, Bryan A. Bassig, Yiming Zhang, Ke Hu, Shunqing Xu, Guanghui Dong, Tongzhang Zheng, Shaoping Yang

**Affiliations:** 1 Wuhan Medical & Healthcare Center for Women and Children, Wuhan, Hubei Province 430030, China; 2 College for Public Health and Social Justice, Saint Louis University, Saint Louis, MO, USA; 3 Wuhan Environmental Monitoring Center, Wuhan, Hubei Province 430000, China; 4 Department of Environmental Health Sciences, Yale School of Public Health, New Haven, CT 06510, USA; 5 Key Laboratory of Environment and Health, Ministry of Education & Ministry of Environmental Protection, and State Key Laboratory of Environmental Health, School of Public Health, Tongji Medical College, Huazhong University of Science and Technology, Wuhan 430030, China; 6 Department of Environmental Health, School of Public Health, Sun Yat-Sen University, Guangzhou 510080, China; 7 Department of Epidemiology, School of Public Health, Brown University, Providence, RI, USA

## Abstract

The objective of this study was to evaluate whether high levels of maternal exposure to O_3_, SO_2_, NO_2_, CO are related to increased risk of congenital heart defects (CHDs) in Wuhan, China. The study included mothers living in the central districts of Wuhan during pregnancy over the two-year period from June 10, 2011 to June 9, 2013. For each study participant, we assigned 1-month averages of O_3_, SO_2_, NO_2_ and CO exposure based on measurements obtained from the nearest exposure monitor to the living residence of mothers during their early pregnancy period. In one-pollutant model, we observed an increased risk of CHDs, ventricular septal defect (VSD), and tetralogy of fallot (TF) with increasing O_3_ exposure. In two-pollutant model, associations with all CHDs, VSD, and TF for O_3_ were generally consistent compared to the models that included only O_3_, with the strongest aORs observed for exposures during the third month of pregnancy. We also observed a positive association between CO exposures during the third month of pregnancy and VSD in two pollution model.Our results contribute to the small body of evidence regarding air pollution exposure and CHDs, but confirmation of these associations will be needed in future studies.

An increasing number of epidemiologic studies have examined whether air pollution exposure is associated with risk of adverse birth outcomes^1^,^2^. Some studies have found strong evidence for an association between exposure to air pollution and infant mortality, particularly postneonatal respiratory mortality and low birth weight^3^,^4^,^5^. In addition, some studies have found that exposure to air pollutants during specific time periods during pregnancy are related to some cardiac anomalies^6^,^7^,^8^,^9^,^10^. However, the evidence is limited, and previous epidemiological studies have suggested conflicting results, which may due to the limited number of studies as well as bias and confounding, chance findings, and limitations in exposure assessment.

Congenital heart defects (CHDs) are the most common severe congenital anomalies and are the leading cause of infant mortality due to congenital anomalies^11^. The precise aetiology of most congenital anomalies is suggested to be multifactorial, with environmental exposures such as air pollution suspected to have a role^12^. Possible mechanisms for the teratogenicity of air pollutants may manifest through the cardiovascular system involving oxidative stress, inflammation, coagulation, endothelial function, hemodynamic responses^12^,^13^, and indirect effects through maternal immune effects^3^. These mechanisms provide biological rationale for the evaluation of the relationship between exposure to air pollution and congenital heart defects.

A recent meta-analysis that combined the results from four individual studies reported that NO_2_ was significantly associated with coarctation of the aorta^8^. Another meta-analysis that combined results from four studies reported associations between NO_2_ exposure and Tetralogy of Fallot (TF), and between SO_2_ exposure and coarctation of the aorta^14^. However, the summary risk estimates were based on a small number of studies, and few specific types of congenital heart defects were explored. Compared to those literatures published in the United States and Europe, a very limited study has been published on the association between ambient air pollution and CHDs in Asia. And almost all prior studies were conducted in developed countries, which may have lower pollution levels and hence narrower ranges of exposure. In contrast, very few studies have been conducted in developing countries where air pollution is usually high (Table 1).

We conducted a cohort study in Wuhan, China, Asia and developed countries to examine the association between maternal exposure to ambient air pollutants and congenital anomalies. Our study included a large, geographically defined population of 105,988 births among women in Wuhan, one of the most polluted cities in China.

## Methods

### Study population

This study used a population-based cohort design. All births delivered between June 10, 2011 to June 9, 2013 were abstracted from a perinatal health care system from Wuhan Medical & Healthcare Center for Women and Children (WMHCWC), which was one of the first three centers in China to standardize its women and children’s health information system. The perinatal health care system is a standardized, computer-based database that includes data collected prospectively on all births, and has accrued approximately 100,000 annual births from nearly all maternity units in Wuhan since its start in 2003. In addition, the database includes information on other characteristics including maternal age, education, parity, infant sex, and season of conception. Births enrolled in our study included live-born infants, stillbirths, and fetal deaths. The study only included mothers living in the central districts of Wuhan during pregnancy. A total of 105,988 admissions and 188 congenital heart defects were collected by our monitoring systems during the study period.

We included cases of selected congenital anomalies among live births, stillbirths after 20 weeks of gestation, as well as pregnancies that were terminated following a prenatal diagnosis of either isolated or multiple congenital heart defects. Cases were diagnosed from clinical, surgical, or autopsy reports, and were coded according to the International Classification of Diseases, 10^th^ Revision (ICD-10). We included in this study only anomaly types that are well defined and recorded, specifically all congenital heart defects combined (Q20–Q28) and the two most common subgroups of cardiac anomalies individually, namely VSD (Q21.0) and TF (Q21.3). Cases with chromosomal anomalies were ineligible for the study, as these may be associated with congenital anomalies of interest in this study and are unlikely to be related to air pollution exposure^15^.

The study protocol was reviewed and approved by the Health Department of Hubei Province and the Institutional Review Board at Wuhan Women and Children Health Care Center.

### Maternal exposure assessment

We used ambient air monitoring data for carbon monoxide (CO), nitrogen dioxide (NO_2_), ozone (O_3_), and sulfur dioxide (SO_2_), which was collected by the Wuhan Environmental Monitoring Center at nine national ambient air quality automatic monitoring stations. Hourly readings were obtained for CO, NO_2_, SO_2_ and O_3_. A daily average was calculated for CO, NO_2_ and SO_2_, whereas an 8-hour average was calculated for O_3_. All pollutants were monitored at all 9 national stations for the entire study period. The stations were located predominantly in the central districts of Wuhan. The collection of air pollutants and installation of air quality monitoring stations was in strict accordance with the monitoring rules on environmental air quality in China, which is a multi-dimensional environmental standard system. It includes quality standards, emission standards, monitoring standards, management standards, and basic standards^16^. For the rules of selection of ambient air quality monitoring stations, it dictates that monitoring stations should not be located directly near traffic and other large emitters (eg industrial sources, incinerators etc) and were mandated not to be influenced by local air pollution in general. Thus the data from the monitoring stations should reflect the general background urban air pollution level rather than local sources (eg, traffic or industrial combustion)^17^,^18^. Concentrations of CO, NO_2_, O_3_, and SO_2_ were assigned to each maternal residence based on the nearest monitor to the residential address reported at the time of the first routine physical examination.

Concentrations of CO, NO_2_, O_3,_ and SO_2_ were assigned to each maternal residence based on the nearest monitor to the residential address reported at the time of the first routine physical examination. By using the estimated date of conception, 14 days from the recorded last menstrual period (LMP), the exposure assessment was performed for the first three months of pregnancy, and we averaged the 24-hr or 8-hr measurements for the first three months of pregnancy. We assigned 1-month averages of the daily values for CO, NO_2_, O_3_ and SO_2,_ and assigned 1-week averages of the daily values for O_3_ for each study participant. We restricted our population to those pregnancies with data available on ≥10 days of each month of the first trimester. This resulted in 97.5–98.9% of the original population for CO, NO_2_ SO_2_ and O_3_. The median distance between the closest monitoring station and the mother’s residence community center was 3.1 KM (0.1 KM–6.0 KM) for CO, NO_2_, O_3_ and SO_2_.

### Statistical methods

Multivariable logistic regression analyses were used to estimate the adjusted ORs and 95% confidence intervals (CI) for the association between exposure to the air pollutants and the risk of congestive heart defects. Based on existing literature and the characteristics of the study population^9^,^11^,^19^,^20^, we consider the variables listed below as major potential confounders and/or effect modifiers: maternal age (<25, 25–35, and >35 years), education (<12, 12, 13–15, >15 years), parity (1, >1), infant sex (male, female), and season of conception (Spring: March–May; Summer: June–August; Fall: September–November; and Winter: December–February).

We constructed one-pollutant models to explore associations between individual pollutants using the monthly exposure assessment and risk of CHDs overall, VSD, and TF. Evaluation of other individual defects was not possible because of the small sample sizes. We conducted two-pollutant models to tease out the effects between the regional pollutants (O3 and SO2) and local pollutants (CO and NO2), without concerning on potential confounding by co-pollutants. Finally, we fitted two-pollutant models with O_3_ and another pollutant. The two-pollutant models provide estimates of the independent effects of CO, NO_2_, SO_2_, and O_3_ on CHD controlling for the second pollutant in the model. We also explored the associations with these outcomes for weekly O_3_ exposure levels (up to 12 weeks) because of uncertainty regarding specific windows of susceptibility and the lack of clearly elucidated mechanisms by which cardiac development could be disrupted by exposure to air pollution^21^.

We present the effect of each pollutant on the risk of CHDs as aORs per a 10-μg/m^3^ change for O_3_, NO_2,_ and SO_2_ and per 100-μg/m^3^ change for CO, along with their 95% CIs. Pearson correlation coefficients were calculated to measure the associations between two air pollutants. Analyses were conducted using SAS 9.3(SAS Institute Inc., Cary, North Carolina) and P < 0.05 was considered statistically significant.

## Results

### Characteristics of the subjects

The distribution of demographic factors of the birth cohort is presented in Table 2. There were 105,988 births during the study period that met the study inclusion criteria. The CHD rate was 17.7 per 10,000, with the highest rate observed for VSD (6.2 per 10,000) followed by TF (2.7 per 10,000). The majority of the cohort members had a maternal age less than 25 years at delivery (69%), and had at least a high school education (85%). For approximately 80% of women, this was their first pregnancy.

### Air pollution and the risk of CHDs

The mean (25^th^ to 75^th^ percentile range) of the exposure concentrations of the air pollutants was 72.41 μg/m^3^ (31.97–106.00 μg/m^3^) for O_3,_ 38.54 μg/m^3^ (18.00–53.91 μg/m^3^) for SO_2,_ 59.61 μg/m^3^ (38.00–77.00 μg/m^3^) for NO_2,_ and 1024.86 μg/m^3^ (701.00–1271.00 μg/m^3^) for CO (Table 1).

Pearson correlation coefficients were calculated for the monthly average concentrations of the air pollutants during the first trimester of pregnancy. NO_2_ and SO_2_, NO_2_ and CO, and SO_2_ and CO were strongly correlated (r = 0.68, r = 0.71 and r = 0.64, respectively). O_3_ was less strongly and negatively correlated with NO_2_, SO_2_ and CO (r = −0.12, r = −0.16 and r = −0.20, respectively).

Table 3 shows the aORs and 95% CIs for the risk of CHDs in relation to O_3_, NO_2_, CO, and SO_2_ exposure by each month of the first trimester of pregnancy. We observed consistently increased risks and dose-response patterns for the risk of CHDs overall and VSD and TF individually in relation to O_3_ exposure levels, and the risk increased gradually as the month of pregnancy increased. For CHDs overall, the adjusted OR for a 10-μg/m^3^ change in O_3_ was 1.06 (95% CI: 1.00–1.13) for the first month of pregnancy, 1.10 (95% CI: 1.03–1.17) for the second month of pregnancy, and 1.12 (95% CI: 1.05–1.19) for the third month of pregnancy. For TF, we observed positive associations in the second month of pregnancy (adjusted OR = 1.24 per 10 μg/m3 change; 95% CI: 1.07–1.44), and third month of pregnancy (adjusted OR = 1.31; 95% CI: 1.13–1.51). The effect estimate for O_3_ exposure for TF during the first month of pregnancy was slightly elevated but not statistically significant (adjusted OR = 1.15; 95% CI: 0.99–1.33). For VSD, a positive association for O_3_ was observed in the third month (adjusted OR for a 10-μg/m3 change in O_3:_ 1.17 (95% CI: 1.05–1.31). The association between O_3_ exposure and VSD during the first and second month of pregnancy was slightly elevated but not statistically significant (adjusted OR = 1.07; 95% CI: 0.96–1.18; adjusted OR = 1.09; 95% CI: 0.97–1.21, respectively). In contrast, higher SO_2_ exposure was associated with decreased risk of CHDs overall in the second and third months of pregnancy. We found no associations between NO_2_ or CO and the selected birth defects.

Table 4 shows the estimated aORs and 95% CIs for the O_3_ weekly exposure analyses in relation to risk of CHDs overall and for VSD and TF individually. Risk of CHDs overall or for VSD and TF showed variability across the first 12 weeks of pregnancy. Specifically, O_3_ exposures after the first 5 weeks of pregnancy, particularly during weeks 8–12, were associated with a greater susceptibility to developing CHDs overall and for VSD and TF. During weeks 8 to 12 (6^th^ week to 10^th^ week after fertilization), the aORs for CHDs overall per 10-μg/m^3^ change in O_3_ exposure ranged from 1.05 to 1.08. For TF and VSD, the corresponding aORs ranged from 1.12 to 1.18 and from 1.10 to 1.12, respectively.

In the two pollutant models (Table 5), a positive association was observed between CO exposures during the third month of pregnancy and VSD (adjusted OR per 100-μg/m^3^ = 1.18; 95% CI: 1.02–1.36). Associations with all CHDs, VSD, and TF for O_3_ were generally consistent in the two pollutant models compared to the models that included only O_3_, with the strongest aORs observed for exposures during the third month of pregnancy. The results of the positive associations for O_3_ in the single pollutant models remained in the two-pollutant models, indicating that observed O_3_ effects were unlikely affected by both regional pollutants and local pollutants.

## Discussion

During the past few decades, CHDs are the most common severe congenital anomalies and the leading cause of infant mortality due to congenital anomalies, and the aetiologies are unknown for the majority of these defects^22^. Recently, studies conducted in developed countries linked ambient air pollution exposure to risk of CHDs; however, these studies provide inconsistent evidence of an association between maternal exposure to ambient air pollutants and CHDs, and few studies have been conducted in developing countries that have higher levels of air pollution^23^.

In this large cohort study conducted among Chinese women and infants exposed to a very high level of pollution, we found that the risk of several CHDs were higher among women with greater exposures to criteria air pollutants. We observed increasing associations between O_3_ exposure and CHDs overall, and VSD and TF individually, and the risk increased gradually as the month of pregnancy increased. The results are consistent to other studies that have explored the associations between O_3_ and CHDs. A previous study conducted in Southern California demonstrated a higher risk of aortic artery and valve defects in relation to increasing O_3_ exposure during the second month of pregnancy^9^. A study conducted in Brisbane used average exposures over the 3–8^th^ weeks of pregnancy and found that O_3_ exposures were associated with an increased risk of pulmonary artery and valve defects when restricted to those women living within 6 kilometers of an ambient air quality monitor^24^. Similarly, a study in Northeast England reported an increased risk of congenital malformations of pulmonary and tricuspid valves with Oxposure after limiting to those living within 16 kilometers of a monitoring station^25^. Other studies detected no positive associations between O_3_ and cardiac defects^11^,^19^,^21^,^26^. A meta-analysis of ambient air pollution and risk of congenital anomalies examined ventricular septal defects and atrial septal defects, but showed no association with O_3_ exposure^8^.

O_3_ is homogenously distributed in areas. And it is a photo- chemical pollutant formed by the reactions of volatile organic compounds and nitrogen oxidesin the presence of sunlight^27^. The possible mechanisms and causes underlying the cardiac development are still unclear, but one potential etiologic pathway indicates that oxidative stress induced by air pollution may affect the migration and differentiation of organogenesis and neural crest cells^6^, which play an important role in heart development^28^. Oxidative stress may also regulate the hemodynamic responses, pulmonary and placental inflammation, and thus the transportation of nutrients and transplacental oxygenation^6^. Ozone is also a powerful oxidizing agent and a highly reactive molecule that may contribute to oxidative stress^13^. This suggests that ozone exposure may have an effect on cardiac development.

The main source of CO in urban area is vehicle exhaust emissions. The number of motor vehicles in urban China are soaring along with the rapid socioeconomic development, and the exhaust emissions are becoming one of the major contributors to urban air pollution^29^. Maternal CO exposure was not associated with CHDs in our study. The results are similar to other studies that have explored the associations between CO and CHDs. Studies conducted in Barcelona^6^, England^3^,^25^, and Israel^11^ reported no association between CO concentrations and cardiac defects. However, some studies have reported positive associations between CO and specific outcomes including ventricular septal defects^9^,^19^, cardiac septa malformations and congenital pulmonary valve stenosis^19^. One study has also reported an inverse association between CO and secundum atrial septal defects^30^. In a meta-analysis of ambient air pollution and risk of congenital anomalies, ventricular septal defects and atrial septal defects were examined, but showed no association with CO exposure^8^. Experimental studies have suggested mechanisms for the fetotoxic effects of CO including reaction with hem-containing proteins, hypoxia, and a reduction in metabolization of xenobiotics^19^. The inconsistent evidence of an association between CO and CHDs need further research.

NO_2_ is a secondary pollutant which is usually less impacted by regional pollution sources^31^. Some studies have found a positive association between NO_2_ exposure and low birth weight, Intra-uterine growth retardation, preterm birth, and stillbirth^32^,^33^,^34^,^35^,^36^. However, epidemiological evidence linking maternal NO_2_ exposure to CHDs is still inconsistent. Studies conducted in California^30^,^37^,^38^, Israel^11^, England^3^, Atlanta^26^, and Texas^20^ reported no association between NO_2_ concentration and cardiac defects. However, the Barcelona study reported a positive association between NO_2_ and coarctation of the aorta using spatial and spatiotemporal exposure models during weeks 3–8 of pregnancy^6^.

SO_2_ emissions have been previously associated with coal in power plants (contributing 49% in 1996 and 59% in 2010 to the total emissions in China) and industrial facilities (34%)^39^,^40^. Maternal SO_2_ exposure was not associated with TF in our study, which is similar with Atlanta study^26^ that have explored the associations between SO_2_ and CHDs. However, other two studies reported inconsistent results. The Northeast England study found an inverse relationship with TF and SO_2_ 19. And the England reported an increased risk of TF due to exposure to SO_2_ 3. For VSD, our study also showed a non-significant relationship between maternal exposure to SO_2_ and VSD. Similarly, other three studies conducted in Atlanta^26^, Brisbane^23^, and nine-states of U.S. 21 were consistent in showing non-significant associations, and one study conducted in Northeast England detected inverse associations^19^. In contrast, the Texas study^20^ and Italy study^41^ have reported an increased risk of VSD due to maternal exposure to SO_2_. Additionally, the Brisbane study also reported an increased risk of aortic artery and valve defects due to SO_2_ exposure^23^. Our findings did not evaluate this association for the small sample, and other four studies also did not support this association^20^,^25^,^26^,^42^. We also found inverse associations between CHDs overall and SO_2_ exposure at the second and third months.

The inverse associations between air pollution exposure and CHDs might be due to chance, but they also might be explained partially by the hypothesis that environmental insults may affect the survival of affected fetuses^19^,^43^. Shaw GM *et al*. have found inverse associations between active smoking and neural tube defects, which may be due to earlier abortion of the fetus^44^. Ritz *et al*. suggested inverse association between CO exposure and chromosomal abnormalities, which may also be explained by the increased vulnerability caused by CO and the resulting increased proportion of early spontaneous abortions^9^.

Most previous studies selected exposure windows to coincide with the period of cardiac morphogenesis and assigned exposures by averaging daily pollutant averages over the critical window (weeks 3–8)^6^,^11^,^15^,^19^,^30^. However, susceptibility windows for these adverse effects arising from environmental insults may not directly coincide with the established stages of fetal heart development^21^, and it is possible that exposures earlier or later in pregnancy could have affected the development of certain malformations^26^. For example, the National Birth Defects Prevention Study conducted in the U.S found that exposure to air pollutants during weeks 2 of pregnancy were associated with risk of pulmonary valve stenosis (PVS)^7^. In our study, the results were sensitive to exposures during specific windows of exposure, and we observed maternal exposures to O_3_ during week 5 and weeks 8–12 of pregnancy were associated with CHDs overall, VSD, and TF.

It is possible that inconsistent findings for air pollution exposure and risk of CHDs across studies may be at least partially due to differences in the level and range of pollution experienced in different countries. Compared with ambient air pollution exposure levels from previous published studies conducted in different countries investigating O_3_, SO_2_, NO_2_ and CO and CHDs, our study have the highest level and range of SO_2_ exposure. However, we didn’t observe a significant association between SO_2_ and TF as the England study did, although we nearly have five times greater SO_2_ exposure levels than the England study^3^, which suggests that it is necessary to evaluate the synergistic effect and interaction of other pollutants effect on CHDs, rather than just exploring one kind of pollutants’ affection. Another possibility of inconsistent findings is that the measurement error in air pollution exposure assessment was too great. The previous studies generally use pure temporal approaches^25^, pure spatial modeling^3^, spatiotemporal modeling, or the nearest monitor approach^26^, which all can introduces measurement error because of the distance between the monitor and the subject. Exposure misclassification of timing may arise from using the estimated date of conception, 14 days from the LMP. Because women may not recall LMP date accurately and the LMP may be unreliable. Exposure misclassification of timing may also arise from using residential information for the mother at the time of birth rather than during the first trimester, which is considered to be the critical period for congenital anomalies^30^. Our study used residential information for the mother during the first trimester exposure to reduce exposure measurement error. Additionally, using air pollutants’ concentrations measured from monitoring stations as proxies for personal exposure assumes that air pollution levels are homogeneous across the study areas. There would be exposure misclassification if local pollution sources existed, such as traffic, construction, or other spatially distributed risk factors. Measurement error would also be different for each of the examined pollutants, as their spatial distribution patterns vary quite widely, and it may also arise from not considering the time spent in different micro-environments^44^.

A general limitation of our study approach is that the prevalence of CHDs may be underestimated because of early fetal loss in those with CHDs, or because minor defects may be asymptomatic and undetected among neonates, which could have reduced the number of CHD cases. And we also did not have data on some other variables that could potentially be confounders, such as maternal diabetes and exposure to passive smoking. In addition, using separate models to assess exposures during correlated adjacent exposure windows might make it harder to identify the biologically-relevant critical window of exposure.

## Conclusion

Our results suggest that exposure to increased levels of O_3_ during the first trimester of pregnancy may contribute to the risk of CHDs in Wuhan, China, which is a highly polluted region of the country. Our results contribute to the body of evidence regarding air pollution exposure and CHDs, but confirmation of these associations will be needed in future studies.

## Additional Information

**How to cite this article**: Zhang, B. *et al*. Ozone and Other Air Pollutants and the Risk of Congenital Heart Defects. *Sci. Rep.* **6**, 34852; doi: 10.1038/srep34852 (2016).

## Figures and Tables

**Table 1:** Characteristics of ambient air pollutants based in Wuhan 2011–2013, and findings from published studies investigating ambient air pollution of O_3_, SO_2_, NO_2_ and CO and the occurrence of CHDs.

Study	Study location	Period	Design	Exposure assessment methods	Results	Air Pollutants	Mean	Minimum	25th percentile	Median	75th percentile	Maximum	IQR
The current study	China	2011–2013	Cohort	Average of daily concentration of pollutants measured at nearest monitoring station	O3 exposure and CHDs overall, and VSD and TF individually	O_3_	72.4 μg/m^3^	1 μg/m^3^	31.9 μg/m^3^	63.6 μg/m^3^	106 μg/m^3^	334.3 μg/m^3^	74.1 μg/m^3^
							(36.9 ppb)	(0.5 ppb)	(16.3 ppb)	(32.4 ppb)	(54 ppb)	(170 ppb)	(37.7 ppb)
						SO_2_	38.5 μg/m^3^	2 μg/m^3^	18 μg/m^3^	32.6 μg/m^3^	53.9 μg/m^3^	261 μg/m^3^	35.9 μg/m^3^
							(14.7 ppb)	(0.8 ppb)	(6.9 ppb)	(12.5 ppb)	(20.6 ppb)	(99.6 ppb)	(13.7 ppb)
						NO_2_	59.6 μg/m^3^	7.1 μg/m^3^	38 μg/m^3^	55.8 μg/m^3^	77 μg/m^3^	174 μg/m^3^	39 μg/m^3^
							(31.7 ppb)	(3.8 ppb)	(20.2 ppb)	(29.6 ppb)	(40.9 ppb)	(92.5 ppb)	(20.7 ppb)
						CO	1.0 mg/m^3^	0.02 mg/m^3^	0.71 mg/m^3^	1 mg/m^3^	1.3 mg/m^3^	4.3 mg/m^3^	0.59 mg/m^3^
							(0.91 ppm)	(0.01 ppm)	(0.62 ppm)	(0.85 ppm)	(1.1 ppm)	(3.8 ppm)	(0.48 ppm)
Gianicolo *et al*.^34^	Southern Italy	2001–2010	Case-control	Daily average exposure measured by 3 monitoring stations over week 3-8 of pregnancy	Exposure to the 90th percentile of SO_2_ to be associated with CHDs	SO_2_	2.8μg/m3	—	—	—	—	—	—
Schembari *et al*.^6^	Barcelona	1994–2006	Case-control	Daily spatio-temperal air pollutants estimates over week 3–8 of pregnancy	A significant association between NO_2_ and coarctation of the aorta	NO_2_	—	—	—	55.7 μg/m^3^	—	—	12.2 μg/m^3^
Stingone *et al*.^15^	Nine U.S.states	1997–2006	Case-control	Daily maximum concentrations using the closest air monitor within 50 km to their residence	NO_2_ was associated with coarctation of the aorta and pulmonary valve stenosis.	O_3_	—	—	32.2ppb	42.9ppb	51.8ppb	—	—
						SO_2_	—	—	—	9.7ppb	—	—	
						NO_2_	—	—	—	33.3ppb	—	—	—
						CO	—	—	—	1.16ppm	—	—	—
Agay-Shay *et al*.^11^	Israel	2000–2006	Cohort	Geographic Information System-based spatiotemporal approach with weekly inverse distance weighting modeling	No significant association had been revealed	O_3_	—	0.45ppb	7.8ppb	26.5ppb	39.1ppb	128ppb	—
						SO_2_	—	0.33ppb	1.5ppb	2.1ppb	3.3ppb	51.4ppb	—
						NO_2_	—	0.2ppb	15.6ppb	23.1ppb	32.3ppb	104.5ppb	—
						CO	—	0.15ppm	0.7ppm	0.9ppm	1.3ppm	13.5ppm	
Padula *et al*.^26^	San Joaquin Valley of California	1997–2006	Case-control	Daily average concentration during the first two months from from more than 20 locations with a maximum interpolation radius of 50 km.	No significant association had been revealed	O3(8-h maximum)	—	10.49ppb	29.05ppb	46.94ppb	62.64ppb	91.92ppb	—
						NO_2_	—	2.4 ppb	13.36 ppb	16.81 ppb	20.53 ppb	38.93 ppb	—
						CO	—	0.13 ppm	0.39 ppm	0.52 ppm	0.71 ppm	1.37 ppm	—
Vinikoor-Imler *et al*.^16^	North Carolina	2003–2005	Cohort	Estimated averaged concentration across weeks 3 through 8 for 12 km x 12 km grid.	No significant association had been revealed	O_3_	40.7 ppb	—	—	42.15 ppb	—	—	30.19 ppb
Dadvand *et al*.^19^	Northeast England	1993–2003	Case-control	Weekly average of pollutants at nearest of 6 monitors to maternal residence	No significant association between SO_2_ and CHDs	SO_2_	—	—	17.6 μg/m^3^	—	31.2 μg/m^3^	—	—
Dadvand *et al*.^22^	Northeast England	1985–1996	Case-control	Two-stage spaiotemporal modeling of weekly exposure levels at maternal residence	Exposure to CO to be associated with VS,DCSM and CPVS. NO was associated with TF.	O_3_	—	—	33.2 μg/m^3^	—	42.4 μg/m^3^	—	—
						NO_2_	—	—	29.2 μg/m^3^	—	38.4 μg/m^3^	—	—
						CO	—	—	0.39 mg/m^3^	—	0.64 mg/m^3^	—	—
Dolk *et al*.^3^	England	1991–1999	Cohort	Estimated annual mean of air pollution for 1 km x 1 km grid	A significant association between SO_2_ and TF	SO_2_	—	—	—	7.86 μg/m^3^	—	—	—
						NO_2_	—	—	—	35.11μg/m^3^	—	—	—
Hansen *et al*.^35^	Brisbane, Australia	1997–2004	Case-control	Daily averaged measurements of air pollution at 18 nearest monitoring stations.	O_3_ was associated with an increased risk of pulmonary artery and valve defects, SO_2_ was associated with an increased risk of aortic artery and valve defects	O_3_	25.8 ppb	4.3 ppb	—	—	—	54.4 ppb	—
					SO_2_	1.5 ppb	0 ppb	—	—	—	7.1 ppb	—	
					NO_2_	8.2 ppb	1.4 ppb	—	—	—	22.7 ppb	—	
CO	1.1 ppm	0.02 ppm	—	—	—	7 ppm	—						
Rankin *et al*.^36^	Northern region, UK	1985–1990	Case-control	Daily average concentration during the first trimester from 62 monitors within 10 km of the mother’s residence.	No significant association between SO_2_ exposure and CHDs	SO_2_			2.7 μg/m^3^		4.4 μg/m^3^		
Strickland *et al*.^35^	Atlanta, USA	1986–2003	Cohort	Average of daily measuremtns of pollutants from one central monitoring station	No significant association between SO_2_, O3, NO2, and CO exposure and CHDs	O_3_	—	—	—	—	—	—	29.9 ppb
						SO_2_	—	—	—	—	—	—	4.0 ppb
						NO_2_	—	—	—	—	—	—	5.7 ppb
						CO	—	—	—	—	—	—	0.3 ppm
Ritz *et al*.^9^	California, USA	1987–1993	Case-control	Average of 24-hr measurements of pollutants at nearest monitoring station	Second-month CO exposure was associated with an increased risk of VSD	O_3_	—	—	1.06 pphm	1.94 pphm	2.84 pphm	—	—
						CO	—	—	1.14 ppm	1.6 ppm	2.47 ppm	—	—

**Table 2:** Characteristics of the study subjects (n = 105, 988).

Item	Infants without any malformations (N = 105,800)	Congentital heart defects (N = 188)
Maternal age (years)		
<20	19813 (18.73)	29 (15.43)
20–25	53060 (50.15)	102 (54.26)
25–30	25237 (23.85)	46 (24.47)
>30	7690 (7.27)	11 (5.85)
Maternal education (years)		
<12	15578 (14.76)	31 (16.49)
12–15	45917 (43.51)	78 (41.49)
>15	44042 (41.73)	79 (42.02)
Missing	263	
Parity		
1	81098 (76.65)	144 (76.60)
>1	24702 (23.35)	44 (23.40)
Season of conception		
Spring	25662 (24.26)	38 (20.21)
Summer	25042 (23.67)	47 (25.00)
Autumn	26833 (25.36)	46 (24.47)
Winter	28263 (26.71)	57 (30.32)
Infant sex		
Male	56355 (53.27)	97 (52.43)
Female	49437 (46.73)	88 (47.57)
Type of birth		
Live birth	105421 (99.64)	29 (15.42)
Stillbirth	372 (0.35)	159 (84.57)
fetal deaths	7 (0.01)	

**Table 3:** Adjusted ^a^ odds ratios and 95% CI of CHD and exposure to O_3_, NO_2_, NO, SO_2_, CO of first 3 months of pregnancy in single pollutant model.

		All congenital heart defects (Q20–Q28)(N = 188)	Ventricular septal defect (Q21.0) (N = 63)	Tetralogy of Fallot (Q21.3) (N = 29)
		aOR [95% CI]	aOR [95% CI]	aOR [95% CI]
O_3_	1^st^ M^b^	1.06 (1.00–1.13)	1.07 (0.96–1.18)	1.15 (0.99–1.33)
	2^nd^ M^c^	1.10 (1.03–1.17)	1.09 (0.97–1.21)	1.24 (1.07–1.44)
	3^rd^ M^d^	1.12 (1.05–1.19)	1.17 (1.05–1.31)	1.31 (1.13–1.51)
NO_2_	1^st^ M^b^	0.90 (0.79–1.02)	0.83 (0.67–1.03)	0.72 (0.53–1.03)
	2^nd^ M^c^	0.94 (0.84–1.06)	0.89 (0.73–1.09)	0.81 (0.60–1.11)
	3^rd^ M^d^	0.90 (0.81–1.01)	0.91 (0.76–1.09)	0.80 (0.60–1.06)
SO_2_	1^st^ M^b^	0.92 (0.81–1.04)	0.90 (0.72–1.12)	0.91 (0.67–1.24)
	2^nd^ M^c^	0.87 (0.76–0.99)	0.82 (0.65–1.03)	1.07 (0.78–1.46)
	3^rd^ M^d^	0.83 (0.73–0.95)	0.81 (0.64–1.02)	0.97 (0.68–1.38)
CO	1^st^ M^b^	0.97 (0.90–1.06)	0.99 (0.86–1.14)	1.01 (0.81–1.26)
	2^nd^ M^c^	0.92 (0.84–1.01)	0.93 (0.81–1.07)	0.97 (0.77–1.21)
	3^rd^ M^d^	0.99 (0.91–1.08)	1.12 (0.87–1.29)	1.05 (0.85–1.30)

^a^Adjusted for maternal age, education, parity, infant sex, season of conception.

^b^1st M = The first month exposure;

^c^2nd M = The second month exposure;

^d^3rd M = The third month exposure.

**Table 4:** Adjusted^a^ odds ratios and 95% CI for CHDs in relation to exposure to O_3_ during the first 12 weeks of pregnancy.

	All congenital heart defects (Q20–Q28)(N = 188)	Ventricular septal defect (Q21.0) (N = 63)	Tetralogy of Fallot(Q21.3) (N = 29)
	aOR [95% CI]	aOR [95% CI]	aOR [95% CI]
1 week	1.04 (0.99–1.09)	1.04 (0.97–1.12)	1.04 (0.94–1.16)
2 week	1.01 (0.97–1.06)	1.07 (0.99–1.14)	1.03 (0.92–1.15)
3 week	1.03 (0.98–1.07)	1.04 (0.97–1.12)	1.04 (0.94–1.16)
4 week	1.02 (0.98–1.07)	1.00 (0.93–1.08)	1.08 (0.97–1.19)
5 week	1.05 (1.00–1.10)	1.05 (0.97–1.13)	1.18 (1.06–1.31)
6 week	1.05 (0.99–1.10)	1.05 (0.96–1.14)	1.06 (0.94–1.19)
7 week	1.03 (0.98–1.09)	1.04 (0.96–1.14)	1.11 (0.99–1.24)
8 week	1.08 (1.02–1.13)	1.03 (0.94–1.13)	1.18 (1.05–1.32)
9 week	1.07 (1.02–1.13)	1.10 (1.01–1.19)	1.18 (1.05–1.31)
10 week	1.07 (1.02–1.12)	1.10 (1.01–1.20)	1.12 (1.01–1.26)
11 week	1.07 (1.02–1.12)	1.10 (1.01–1.16)	1.13 (1.01–1.27)
12 week	1.05 (1.00–1.10)	1.12 (1.04–1.21)	1.12 (1.01–1.25)

^a^Adjusted for maternal age, education, parity, infant sex, season of conception.

**Table 5:** Adjusted^a^ odds ratios and 95% CI of CHD and exposure to O_3_, NO_2_, NO, SO_2_, CO of first 3 months of pregnancy in two pollutant model.

		All congenital heart defects (Q20–Q28)(N = 188)	Ventricular septal defect (Q21.0)(N = 63)	Tetralogy of Fallot (Q21.3) (N = 29)
		aOR [95% CI]	aOR [95% CI]	aOR [95% CI]
O3 + NO2				
O3	1st M^b^	1.05 (0.98–1.12)	1.03 (0.92–1.16)	1.09 (0.92–1.29)
	2nd M^c^	1.10 (1.03–1.17)	1.07 (0.96–1.20)	1.23 (1.05–1.43)
	3rd M^d^	1.11 (1.04–1.19)	1.16 (1.03–1.30)	1.29 (1.11–1.50)
NO2	1st M^b^	0.94 (0.82–1.08)	0.86 (0.67–1.10)	0.79 (0.55–1.13)
	2nd M^c^	0.97 (0.86–1.10)	0.91 (0.74–1.12)	0.89 (0.65–1.22)
	3rd M^d^	0.95 (0.85–1.06)	0.97 (0.80–1.16)	0.93 (0.69–1.25)
O3 + SO2				
O3	1st M^b^	1.05 (0.99–1.12)	1.05 (0.94–1.18)	1.15 (0.98–1.34)
	2nd M^c^	1.08 (1.01–1.16)	1.06 (0.94–1.19)	1.27 (1.09–1.49)
	3rd M^d^	1.11 (1.04–1.18)	1.15 (1.03–1.29)	1.31 (1.14–1.52)
SO2	1st M^b^	0.94 (0.83–1.07)	0.93 (0.73–1.18)	0.99 (0.71–1.36)
	2nd M^c^	0.90 (0.79–1.03)	0.84 (0.66–1.06)	1.19 (0.86–1.65)
	3rd M^d^	0.86 (0.75–0.98)	0.84 (0.66–1.06)	1.08 (0.75–1.54)
O3 + CO				
O3	1st M^b^	1.06 (1.00–1.13)	1.07 (0.96–1.19)	1.16 (1.00–1.35)
	2nd M^c^	1.09 (1.02–1.16)	1.07 (0.96–1.20)	1.25 (1.07–1.45)
	3rd M^d^	1.13 (1.06–1.20)	1.20 (1.07–1.33)	1.32 (1.15–1.52)
CO	1st M^b^	1.00 (0.92–1.09)	1.02 (0.88–1.18)	1.08 (0.86–1.35)
	2nd M^c^	0.94 (0.86–1.03)	0.95 (0.82–1.10)	1.02 (0.82–1.27)
	3rd M^d^	1.03 (0.94–1.12)	1.18 (1.02–1.36)	1.13 (0.91–1.41)

^a^Adjusted for maternal age, education, parity, infant sex, season of conception.

^b^1st M = The first month exposure;

^c^2nd M = The second month exposure;

^d^3rd M = The third month exposure.
